# HIV-1 drug resistance genotyping success rates and correlates of Dried-blood spots and plasma specimen genotyping failure in a resource-limited setting

**DOI:** 10.1186/s12879-022-07453-9

**Published:** 2022-05-17

**Authors:** Jonah Omooja, Nicholas Bbosa, Dan Bugembe Lule, Maria Nannyonjo, Sandra Lunkuse, Faridah Nassolo, Stella Esther Nabirye, Hamidah Namagembe Suubi, Pontiano Kaleebu, Deogratius Ssemwanga

**Affiliations:** 1 Medical Research Council/Uganda Virus Research Institute and London School of Hygiene and Tropical Medicine, Uganda Research Unit, Entebbe, Uganda; 2 Uganda Virus Research Institute, Entebbe, Uganda; 3 London School of Hygiene and Tropical Medicine, London, UK

**Keywords:** HIV-1, Genotypic resistance testing, Success rates, DBS, Plasma, Resource-limited settings

## Abstract

**Background:**

HIV-1 drug resistance genotyping is critical to the monitoring of antiretroviral treatment. Data on HIV-1 genotyping success rates of different laboratory specimen types from multiple sources is still scarce.

**Methods:**

In this cross-sectional study, we determined the laboratory genotyping success rates (GSR) and assessed the correlates of genotyping failure of 6837 unpaired dried blood spot (DBS) and plasma specimens. Specimens from multiple studies in a resource-constrained setting were analysed in our laboratory between 2016 and 2019.

**Results:**

We noted an overall GSR of 65.7% and specific overall GSR for DBS and plasma of 49.8% and 85.9% respectively. The correlates of genotyping failure were viral load (VL) < 10,000 copies/mL (aOR 0.3 95% CI: 0.24–0.38; p < 0.0001), lack of viral load testing prior to genotyping (OR 0.85 95% CI: 0.77–0.94; p = 0.002), use of DBS specimens (aOR 0.10 95% CI: 0.08–0.14; p < 0.0001) and specimens from routine clinical diagnosis (aOR 1.4 95% CI: 1.10–1.75; p = 0.005).

**Conclusions:**

We report rapidly decreasing HIV-1 genotyping success rates between 2016 and 2019 with increased use of DBS specimens for genotyping and note decreasing median viral loads over the years. We recommend improvement in DBS handling, pre-genotyping viral load testing to screen samples to enhance genotyping success and the development of more sensitive assays with well-designed primers to genotype specimens with low or undetectable viral load, especially in this era where virological suppression rates are rising due to increased antiretroviral therapy roll-out.

## Importance

Genotypic resistance testing is crucial to HIV treatment as it guides on treatment decisions such as when to switch to which HIV regimens for individuals on HIV antiretroviral therapy. This is vital in achieving HIV virological suppression, a key outcome in minimizing the transmission of HIV. In resource-constrained settings, the use of DBS specimens has been popularised to circumvent logistical challenges associated with plasma, but genotyping success rates (GSR) of DBS are frustrating. We report lower GSR of DBS compared to plasma specimens and note that low GSR were associated with viral loads < 10,000 copies/mL, DBS specimens and specimens from routine clinical diagnosis. We highlight that as VLs continue to decrease due to suppression following ART rollout, we need more robust laboratory assays to genotype DBS specimens and emphasize the essence of proper handling of DBS specimens prior to genotyping. These findings are of fundamental public health importance as they may be used to improve laboratory performances in settings like ours and improve HIV treatment monitoring and outcomes.

## Introduction

Sub-Saharan Africa remains the worst hit by the HIV/AIDS epidemic with 20.7 million of the global estimated 38 million HIV-infected individuals by 2020 [1]. A tremendous achievement has been realised in increasing access to antiretroviral therapy (ART) among individuals living with HIV [1]. This is a huge milestone in combating the epidemic since ART suppresses viral replication, reduces the morbidity associated with HIV/AIDS and curbs HIV transmission to uninfected individuals [2]. Increased access to ART comes with a challenge of increasing prevalence of HIV drug resistance (HIVDR) [3, 4] which compromises ART benefits [5]. Monitoring of HIVDR remains a priority in the WHO strategy for combating the HIV/AIDS epidemic. The new UNAIDS 2025 targets call for 95% of HIV-infected individuals becoming aware of their status, initiation of 95% of those with known HIV-positive status on treatment and having 95% of those on ART achieving virological suppression [6, 7]. To achieve these targets, virological monitoring and HIV genotypic resistance testing of individuals on ART are necessary as recommended by the WHO [7]. Viral load testing provides a basis for ART response monitoring and guides the need to switch drug regimen classes [2]. Guidelines in resource-rich settings recommend HIV-infected individuals to undergo viral load testing at ART initiation or modification. Due to transmitted HIVDR, genotypic resistance testing for mutations in the reverse transcriptase and protease genes is recommended prior to ART initiation or modification to guide selection of ART regimen [8]. In contrast, guidelines in resource-constrained settings like Uganda recommend viral load testing for adults 6 months post -ART initiation and subsequently every 12 months for those with viral load suppression. Also, genotypic resistance testing is only recommended for adult individuals failing on 2nd line and 3rd line regimes, and those failing on protease inhibitor-based or dolutegravir-containing regimens [2]. To circumvent economic barriers that impede ART monitoring in resource-constrained settings, the WHO has recommended a relatively cost-effective public health approach for surveillance of acquired [9] and pre-treatment [10] HIVDR in resource limited settings. However, this too, requires HIVDR genotyping.

HIV-1 genotypic testing is a nucleic acid amplification and sequencing test aimed at detecting the existence of HIVDR mutations in targeted regions of the HIV-genome such as protease, reverse transcriptase and integrase genes [11, 12]. It also examines HIV diversity and generates data used for molecular epidemiological and evolutionary studies [13, 14]. The interpretation of HIVDR genotyping data is done by a clinical expert or with the aid of an HIVDR database [15]. HIV genotyping consists of, extraction and purification of the viral nucleic acid, amplification of the target gene and sequencing of the amplicons.

Plasma specimens are preferred to dried plasma/serum and blood spot specimens for HIV genotyping in most research studies and clinical applications [16]. This is because plasma sequence data represents actively-replicating viruses which form a large proportion of viruses circulating in the body [17]. However, the need for cold-chain and associated transportation and storage costs have made plasma less appropriate in resource-constrained settings, especially rural areas. Consequently, dried blood spot (DBS) specimens offer a cheaper alternative to the conventional use of plasma for virological monitoring [18–20] and HIV genotypic resistance testing [21–25]. The preference of DBS to plasma specimens is because DBS specimens are easy to collect, process, transport and can be kept at ambient temperature for relatively longer time and thus do not require a costly and a not-readily available cold-chain in resource-constrained settings [18, 21, 22]. Furthermore, many studies report concordance between paired plasma and DBS specimens for HIV genotyping [21–23, 25, 26], although one study reported discordance based on next-generation sequencing [27]. Genotyping success rates of DBS specimens are, however, continually variable due to several factors.

Following the renewed global efforts to eliminate the public threat posed by HIV/AIDS [6], HIV genotypic drug resistance testing is crucial to the success of ART programmes. The clinical community is reliant on results of HIV genotypic tests in making important clinical decisions such as which drug regimen to offer and when to switch to another regimen class. It is therefore imperative for laboratories to ascertain the success rates of specimens they analyse. Currently in our setting, there is a paucity of data on the genotyping success rates of unpaired DBS and plasma specimens. In the current study, we retrieved the data of DBS and plasma specimens analysed in the MRC/UVRI and LSHTM virology laboratory between 2016 and 2019 and evaluated the genotyping success rates of both DBS and plasma specimens. In addition, we assessed the possible correlates of genotyping failure of both specimen types. This data is vital for quality assurance assessment and is informative in decisions for improving not just the performance of the laboratory assays but also the management of studies and clinical facilities from which specimens are obtained.

## Methods and materials

### Study design and setting

In this cross-sectional study, we analysed the yield of genotyping success, herein referred to as genotyping success rates (GSR), with 6837 unpaired specimens of which 3836 were DBS and 3001 were plasma specimens that were brought to our laboratory for HIVDR genotyping between 2016 and 2019. All these specimens were coming from within Uganda except for cross-border DBS specimens that were shipped at ambient temperatures in sealed envelopes from Malawi. Our standard operation procedures for sample reception were used to accept the specimens (both plasma and DBS). Plasma specimens were accepted if they were correctly labelled, and the labels matched with the accompanying laboratory request forms. DBS specimens were accepted if they came with clear information that matched the paperwork, had at least three blood spots with a dark uniform colour and packed in glassine envelope, placed in a gas-impermeable, zip closure plastic bags [28, 29]. For studies that used plasma specimens, we accepted plasma with sufficient volumes in intact tubes (not leaking), stored at – 80 °C at collection points and transported in liquified nitrogen tanks within 3 days from the collection date [29]. The methods of transportation of laboratory specimens that we followed have been published elsewhere [30].

### Data cleaning and processing

We retrieved sequencing data from our laboratory database and categorised specimen by year of sequencing and specimen type. We defined genotyping success of a specimen as completion of the entire process of specimen preparation, extraction, amplification, and generation of good quality HIV sequences (genotypes). Good quality sequences were those whose chromatograms were evenly-spaced peaks, each representing a single nucleotide and having no or very minimal baseline “noise,” if any [31]. The genotyping success rates refer to the percentage proportions of specimens whose specimens yield good quality nucleotide sequences, with the denominator being the total number of specimens presented for genotyping. Specimens were from different studies and clinical facilities in which individuals ranged from children to adults on ART. The dependent variable of interest was the genotyping result, either success or failure. The independent variables analysed were viral load, specimen type, source of the specimen (research study or routine clinical diagnosis). Research studies strictly adhere to clear standard operating procedures (SOPs) and possess proper facilities to ensure specimen integrity while specimens from routine clinical diagnosis came from health facilities, public clinics/laboratories which are not entirely research focused. As such, some facilities may have less stringent adherence to SOPs and insufficient infrastructure to ensure sample integrity. We excluded specimens whose genotyping results were missing in our database.

### PCR amplification and HIV genotypic drug resistance testing

Viral load tests for both plasma and DBS specimens were done using Roche CoBAS TaqMan^®^ platforms (Roche Molecular Systems, Branchburg, NJ USA) with methods described by Pollack et al. [32]. The modification made for DBS specimens was the incubation of two blood spots incubated for 10 min with 1 mL of sample pre-extraction (SPEX) buffer in a thermomixer set at 1000 rpm. The sample was then processed using the COBAS^®^ AmpliPrep/COBAS^®^ TaqMan^®^ HIV-1 Test v2.0 kit (Roche Molecular Systems, Branchburg, NJ) and the dried fluid spot procedure protocol (H12DFSP96) following the manufacturer’s instructions and as described earlier [32].

Both DBS and plasma specimens received in the laboratory were stored at -80^0^C prior to HIVDR testing.

In processing the DBS specimens, we excised three spots using a DBS puncher, into a 2 mL NucliSENs lysis buffer (Biomereux, Germany), and lysis occurred on a roller mixer for 1 h at room temperature. For plasma specimens, 100 µL of specimen was added into a 2 mL NucliSENS lysis buffer and lysis done as already stated for DBS specimens. For both DBS and plasma specimens, we used a NucliSENS MiniMAG^®^ (Biomerieux, Marcy l’Etoile, France) system to extract total nucleic, eluting 30 µL of RNA following the manufacturer’s instructions and our in-house validated assay. RNA from plasma specimens received in 2016 (before we introduced NucliSENS MiniMAG^®^) had been extracted using the Qiagen kit as previously described [33].

We used 10 µL of RNA for either type of specimens for reverse transcription and complementary DNA (cDNA) synthesis with superscript III high-fidelity one-step PCR kit (Invitrogen), followed by a nested PCR with 2 µL of the cDNA, using a validated inhouse assay [22, 29]. The HIV-1 polymerase sequences of the complete HIV-1 protease gene (1–99 amino acids) and the reverse transcriptase gene (1–320 amino acids) were realised from chromatogram data using either RECall [34] or Sequencher (Gene codes Corporation, Ann Arbor, MI, USA). For purposes of quality control, our laboratory subscribes to the virology quality assurance (VQA) programme: All sequences generated in the laboratory were examined for any possible cross-contamination by creating maximum-likelihood phylogenetic trees with 1000 bootstraps using RaxML [35]. The sequences that passed the HIVDR VQA test were analysed for HIV drug resistance mutations using the Stanford HIVdb [36].

### Statistical analysis

We did statistical analyses using Stata v15 (StataCorp, USA). Medians and interquartile ranges (IQR) were used for continuous variables while frequencies and proportions were used for categorical variables. Univariate logistic regression analysis was done to examine the association of independent variables with the outcomes of interest. Only variables with a p < 0.2 in bivariate analysis were entered to adjusted multivariate logistic regression models. To be considered significantly associated with the outcome, only variables with a p < 0.05 were reported. We presented the results of adjusted odds ratios (aOR) with 95% confidence intervals (CI).

## Results

### Profile of DBS and plasma specimens analysed

Between 2016 and 2019, we tested 6837 specimens for HIV drug resistance, 3836 (56.1%) were DBS while 3001 specimens (43.9%) were plasma. Over this period, the number of specimens generally rapidly increased with a slight decline from 2018 to 2019. The number of DBS specimens exponentially increased over the years while the number of plasma specimens rapidly decreased (Table 1). Overall, research studies contributed 51.7% (3533/6837) while routine clinical diagnosis provided the rest of the specimens received in our laboratory. Most plasma specimens, 75.6% (2670/3533) were from research studies while most of the DBS specimens received, 90% (2973/3304), were from routine clinical diagnosis (Table 1). The increase in DBS was attributed to the roll out of DBS for the national viral load programme and the subsequent use of remnant DBS for HIVDR testing among patients failing ART in Uganda.

**Table 1:** Profile all laboratory specimens analysed between 2016 and 2019

	Overall total	For sample type	
		DBS	Plasma
Number analysed	6837	3836	3001
Proportion (%)	100	56.1	43.9
Successfully genotyped	4489	1910	2579
Genotyping success rate (%)	4489/6837 (65.7)	1910/3836 (49.8)	2579/3001 (85.9)
Year of laboratory analysis N (%)			
2016	1990	89/1990 (4.5)	1901/1990 (95.5)
2017	1001	484/1001 (48.4)	517/1001 (51.6)
2018	1699	1302/1699 (76.6)	397/1699 (23.4)
2019	2147	1961/2147 (91.3)	186/2147 (8.7)
Specimen source			
Research studies	3533	863/3533 (24.4)	2670/3533 (75.6)
Routine clinical diagnosis	3304	2973/3304 (90.0)	331/3304(10.0)
Pre-genotyping viral load testing			
Not done	3893	1884/3893 (48.4)	2009/3893 (51.6)
Done	2944	1952/2944 (66.3)	992/2944 (33.7)
Median viral load for known	4.31log10 (3.57–4.93log10)	4.12log10 (3.43–4.8log10)	4.62 log (3.94–5.13log10)

### Viral load testing and viral load trends

**Fig. 1:**
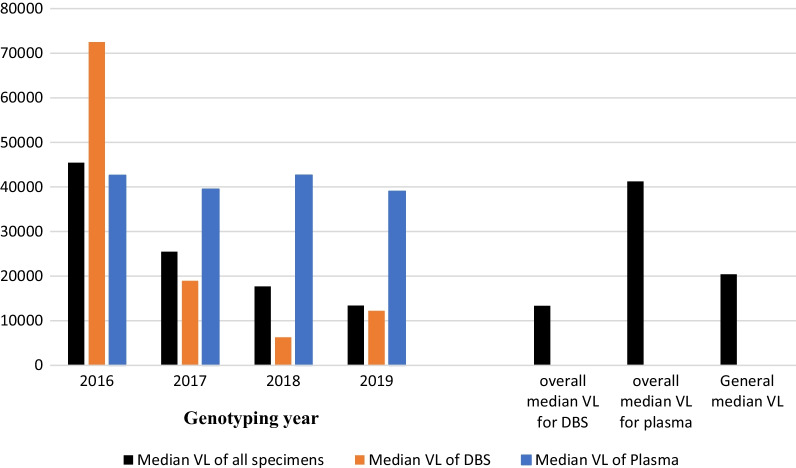
Trends in the median VLs of the specimen genotyped over the years. Median VLs were determined for all specimens analysed in each year. Overall median VLs for DBS and plasma, and General median VLs represent values obtained for specimens over the 4 years

Of the 6837 specimens, pre-genotyping viral load testing had been done for 43.1% (2944/6837) of the specimens (Table 1). Out of 2944 specimens with pre-genotyping viral load testing results, 66.3% (1952/2944) were DBS specimens while the rest were plasma specimens. Each of the DBS specimens with viral load results was shipped with two DBS cards, one for viral load testing and another for genotypic resistance testing. The overall median viral load for all the specimens with prior VL testing results was 4.31 log10 [IQR: 3.57 log10-4.93 log10)]. Over the 4 years, the overall median VL of plasma specimens [4.62 log (IQR: 3.94–5.13 log10)] was higher than for DBS specimens [4.12 log10 (IQR: 3.43–4.8 log10)]. We noted median VLs of the specimens presented for genotyping rapidly decreasing over the years (Fig. 1). This trend corresponds to the increase in the proportion of the DBS specimens (with relatively lower median VLs) analysed.

### Genotyping success rates of HIV DBS and plasma specimens (2016–2019)

**Fig. 2:**
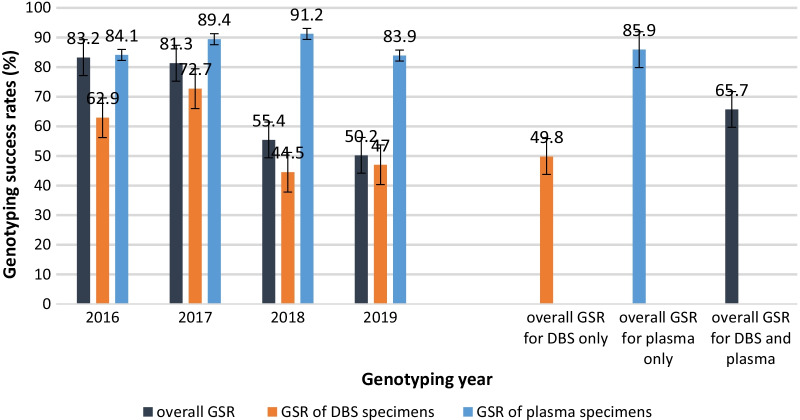
Trends in the overall and genotyping success rates of DBS and plasma specimens from 2016 to 2019. Overall genotyping success rates (GSR) were obtained by analysing all specimens genotyped in the 4 years (2016–2019). The GSR was a proportion of specimens that yielded clean sequences (genotypes) with the denominator being the total number of specimens that we attempted to genotype in that year or in all the 4 years for the overall GSR (see Table 1 for denominator values). The analysis was also independently done for DBS and plasma specimens

We successfully genotyped 4489 specimens between 2016 and 2019, thus an overall GSR of 65.7% for both specimen types over the 4-year period. From 2016 to 2019, the overall GSR (for both specimen types) generally decreased, with the highest overall GSR of 83.2% attained in 2016 and the lowest GSR of 50.2% recorded in 2019 (Fig. 2). We noted an overall DBS GSR of 49.6% which was considerably lower than 85.9% GSR of plasma over the analysis period (Fig. 2). The GSR of DBS specimens also decreased in the same period; from 62.9% in 2016 to 47% in 2019. There was however a gradual increase in GSR of DBS between 2016 and 2017, with highest GSR of 72.7% realised in 2017. Conversely, the GSR of plasma increased gradually from 2016 to 2018 and slightly decreased between 2018 and 2019 (Fig. 2).

### Correlates of HIV-1 genotyping failure

In Table 2, we determined the independent correlates of genotyping failure as low viral loads, DBS specimens, lack of pre-genotyping viral load testing, and genotyping of specimens from routine clinical diagnosis.

**Table 2:** Correlates of genotyping failure for all specimens analysed from 2016 to 2019

Predictor Variable	Genotyping failure n/N (%)	Unadjusted OR	p-value	Adjusted OR	p-value
Specimen type					
DBS	1926/3836 (50.2)	1	< 0.0001		**< 0.0001**
Plasma	422/3001 (14.1)	0.16 (0.14–0.18)		0.10 (0.08–0.14)	
Pre-genotyping viral load testing					
Not done	1398/3893 (35.9)	1	0.002		**0.023**
Done	950/2944 (32.3)	0.85 (0.77–0.94)		0.64 (0.13–0.56)	
Viral load					
1001–10,000 cps/mL	634/1165 (54.4)	1	< 0.0001		**< 0.0001**
> 10,000–50,000 cps/mL	197/755 (25.4)	0.28 (0.23–0.35)		0.3 (0.24–0.38)	
> 50,000–200,000 cps/mL	81/609 (13.3)	0.13 (0.10–0.17)		0.14 (0.11–0.19)	
> 200,000 cps/mL	38/392 (9.7)	0.09 (0.06–0.13)		0.10 (0.07–0.14)	
Specimen source					
Research studies	745/3533 (21.1)	1	< 0.0001		**0.005**
Routine clinical diagnosis	1603/3304 (48.5)	3.5 (3.17–3.92)		1.4 (1.10–1.75)	

Each denominator represents the number of specimens of that specific category

**Fig. 3:**
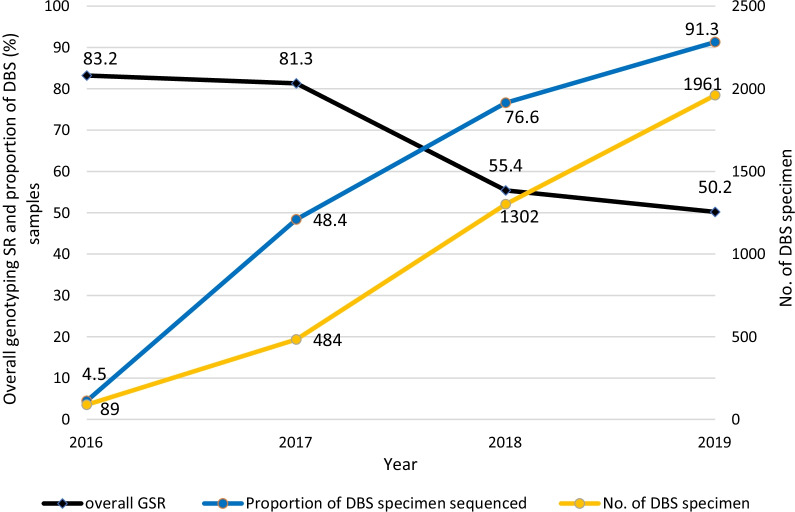
Variations in genotyping success rates, number and proportion of DBS specimens genotyped with time. The proportion of DBS specimens represents the number of DBS specimens being the numerator and the denominator being the total number of specimens we attempted to genotype in a particular year (see Table 1). The overall genotyping success rates (GSR) decreased as both the number and proportion of DBS specimens we attempted to genotype increased from 2016 to 2019

The specimen source was independently associated with genotyping failure. Specimens that originated from routine clinical diagnosis had significantly higher odds of failing the genotypic test compared to research studies (aOR 1.4 95% CI: 1.10–1.75, p = 0.005). In addition, we noted that the DBS specimens were 90% more likely to fail genotypic testing compared to plasma specimens (aOR 0.10 95% CI: 0.08–0.14; p = 0.0001). The overall genotyping success rates (GSR) decreased as both the number and proportion of DBS specimens we tested increased from 2016 to 2019 (Fig. 3).

Specimens analysed without pre-genotyping viral load testing failed genotypic resistance testing more frequently than those where pre-genotyping viral load testing had been done. Lack of pre-genotyping viral load testing significantly increased the odds of genotypic resistance testing by 36% (aOR 0.64 95% CI: 0.13–0.56; p = 0.001) (see Table 2).

Specimens with viral loads < 10,000 copies/mL were more likely to fail a genotyping test compared to those with viral loads > 10,000 copies/mL. Specimens with VL > 10,000–50,000 were 70% less likely to fail genotypic testing than specimens with VL < 10,000 copies/mL (aOR 0.3 (95% CI: 0.24–0.38, p < 0.0001). The odds of failing genotypic testing decreased with increased viral load. Specimens with VL > 200,000copies/mL had only 10% chances of failing a genotypic test (aOR 0.10 (95% CI:0.07–0.14), p < 0.0001) (Table 2).

### Correlates of genotyping failure of DBS specimens

On realising the underwhelming genotypic success rates of DBS specimens, we assessed the possible predictors of genotyping failure and found that low viral loads, lack of pre-genotyping viral load testing and DBS specimens from routine clinical diagnosis correlated with genotyping failure (Table 3).

**Table 3:** Correlates of DBS specimens genotyping failure

Factor	Genotyping failure n (%)	Odds ratio (95% CI)	p-value	Adjusted OR [aOR (95% CI)]	p-value
Pre-genotyping viral load testing					
Not done	1038/1885 (55.1)	1	< 0.0001		**0.001**
Done	888/1952 (45.5)	0.68 (0.6–0.77)		0.71 (0.16–0.22)	
Viral load					
1000–10,000 cps/mL	599/897 (66.8)	1	< 0.0001		**< 0.0001**
> 10,000–50,000 cps/mL	185/497 (37.2)	0.29 (0.23–0.37)		0.3 (0.24–0.38)	
> 50,000–200,000 cps/mL	73/344 (21.2)	0.13 (0.11–0.18)		0.14 (0.10–0.18)	
> 200,000 cps/mL	31/214 (14.5)	0.08 (0.06–0.13)		0.07 (0.06–0.13)	
Specimen source					
Research studies	48/137 (35.0)	1	< 0.0001		**0.006**
Routine clinical diagnosis	1574/3279 (48.0)	1.72 (1.36–2.18)		1.4 (1.11–1.84)	

Each denominator represents the number of specimens of that specific category

DBS specimens that were analysed without pre-genotyping viral load testing had 29% more chances of failing genotyping testing than those DBS specimens with pre-genotyping viral load test results (aOR 0.71 95% CI: 0.16–0.22, p < 0.001).

Low viral loads < 10,000 copies/mL were significantly associated with DBS genotyping failure. We noted that the odds of genotyping failure of DBS specimens significantly decreased with increasing viral load. Specimens with VL > 200,000 copies/mL had the lowest odds of failing genotypic compared to specimens with lower VL (aOR 0.07 95% CI: 0.06–0.13, p < 0.0001).

Genotyping of DBS specimens from routine clinical diagnosis significantly increased the odds of failing the genotypic resistance testing (aOR 1.4 95% CI: 1.11–1.84, p = 0.006).

## Discussion

HIV drug resistance genotyping has revolutionised the clinical management of individuals on ART because it informs decisions like when to implement regimen switch. Successful genotyping in the laboratory is critical to patient care. In this cross-sectional study, we examined the genotyping success rates of unpaired DBS and plasma specimens analysed in a WHO-designated HIVDR laboratory in a resource-constrained setting in Uganda and assessed the correlates of specimen genotyping failure.

From 2016 to 2019, we obtained an overall GSR of 65.7% for all specimens delivered for analysis in our laboratory, but the overall GSR for plasma was higher (85.9%) than that of DBS specimens (49.8%) over that 4-year period. The plasma genotyping success rate in this study was lower than the 100% genotyping success rate that was realised in our previous study [37]. Similarly, the genotyping success rate of DBS specimens reported here is also lower than that reported by Zhang et al. in a survey in Kenya and Tanzania [38]. The comparatively higher genotyping success rates in those studies could be attributed to them being well controlled research studies in which the study staff were well trained, monitored, and specimen collection, transportation and storage done according to well-designed protocols. In contrast, this study had specimens from both research studies and routine clinical diagnosis including cross-border specimens that required lengthy shipping procedures, with most specimens from routine clinical diagnosis contributing significantly to genotyping failure (p < 0.0001). Though not part of our analyses, we have in some cases received DBS specimens 3 months from the collection date. Aware of inadequate facilities in most of these facilities, it is possible that DBS specimens shipped to our laboratory for genotyping were probably not handled under optimum conditions that maintain sample integrity.

Studies have shown that better genotyping success rates are realised when DBS specimens are kept at ambient temperatures for 2 weeks and stored frozen at – 80 °C prior to shipping, and that prolonged storage for over 2 weeks at ambient temperature reduced genotyping success rates [22, 39]. Furthermore, most research studies prepared their DBS cards with venous blood while most routine clinical facilities preferred finger prick DBS. Parry et al. showed that venous DBS had better genotyping success rates compared to finger prick DBS [22]. This probably explains why specimens from research studies had better genotyping success rates. It is probable that the site of collection of blood for DBS specimen preparation could have impacted on the genotyping success rates of the DBS samples. It will be interesting for future studies to assess the various DBS preparation methods and their impact on GSR of DBS specimens.

Generally, the genotyping success rates of DBS specimens were substantially lower than those of plasma over the 4 years. Our analysis revealed that over the 4 years, the overall GSR rapidly decreased with increasing number of DBS specimens presented for genotyping. The observation that the overall GSRs were decreasing in a similar trend as genotyping success rates of DBS specimens, further asserts that DBS specimens, to a large extent, contributed to overall genotyping failure. Our logistic regression analysis showed that DBS were at 90% more odds of genotyping failure compared to plasma specimens (aOR 0.10; 95% CI: 0.08–0.14; p < 0.0001) correlating DBS specimens to genotyping failure. This probably arose from the large proportion of DBS specimens originating from routine clinical diagnosis that face significant challenges in terms of specimen collection, transportation, and storage. Moreover, DBS have considerably gained preference to plasma because DBS specimens are easy to collect, transport and do not require a cold chain [22, 40]. This explains why we received more DBS than plasma specimens between 2017 and 2019. The increase in DBS shipped to our laboratory was also attributed to the roll out of DBS for the national viral load programme and the subsequent use of remnant DBS for HIVDR testing among patients failing ART in Uganda. This popularity should be followed by precautions to ensure that specimen quality is not compromised on the way to the laboratory for genotyping. Evidence from other studies suggests that DBS specimen yield appreciable genotyping rates when specimens are properly handled to ensure nucleic acid integrity [38, 41].

The higher genotyping success rates of plasma compared to DBS specimens reported here mirrors with findings of other researchers [26, 42, 43]. The better GSR of plasma could probably be attributed to a higher rate of HIV-1 RNA degradation for DBS compared to plasma because of the extra processing time at ambient temperature. Temperature and humidity at which DBS specimens are stored affects the stability and integrity of nucleic acids embedded on filter papers [44]. Despite the lower GSR of DBS compared to plasma in our HIVDR genotyping laboratory, we cannot undermine the reliability of DBS specimens as a suitable alternative to plasma especially in resource-constrained settings. Studies by Rottinghaus et al. [45] and Diouara et al. [46] both corroborate the suitability of DBS for HIV drug resistance testing.

For both DBS and plasma specimens, we noted a significant association between VL levels and HIVDR genotyping rates: As expected, VLs < 10,000 copies/mL were associated with genotyping failure (p < 0.0001). A similar observation was also noted when DBS specimen were analysed alone. In studies that examined the use of DBS for genotyping, DBS with VL < 10,000 copies/mL had reduced genotyping success rates [22, 27]. Our findings also concur with studies that cited VL as the major determinant of concordance between plasma and DBS especially when viral load ≥ 5000 copies/mL [21, 25, 27, 47]. Studies analysing paired DBS and plasma specimens report significant correlation and strong concordance in RNA levels between plasma and DBS specimens [48, 49]. Similar to findings in this study, viral loads considerably influenced genotyping success rates in a survey carried out in Kenya and Tanzania where DBS specimens with VLs ≥ 5000 copies/mL achieved genotyping success rate of 90% [38]. Interestingly, in our study, a substantial proportion of specimens had VL < 10,000 copies/mL and could explain why the overall GSR was only 65.7%. This implies that in this era when virological suppression (VL < 1000 copies/mL) rates nearing or exceeding 90% have been observed [37, 41], and following recommendations to use more potent dolutegravir-containing regimens, genotyping success rates are likely to continue to decline. There is evidence in this study that shows median viral loads of specimens analysed in our laboratory rapidly decreasing from 2016 to 2019. This can be explained by rapid scale-up of ART following recommendations of the WHO and subsequent high VL suppression rates reported in our setting [41]. What is more intriguing is that even at VL < 1000 copies/mL, HIVDR variants can still be selected as reported in a Kenyan study in which plasma samples with VL ≤ 1000 copies/mL had a genotyping success rate of 32% [50]. Ultimately, robust techniques need to be adopted to ensure that genotyping assays are adapted to successfully genotype both plasma and DBS specimens at VL < 1000 copies/mL. Some studies have already demonstrated the possibility of successful genotyping at low and undetectable viremia [50–52].

The findings of our study also highlight the importance of virological monitoring. Our findings show that 53.9% of the specimens analysed were brought for genotyping without prior viral load testing. This is expected in our setting where viral load testing is not widely available for all HIV-infected individuals due to high costs. We noted significantly higher odds of genotyping failure in specimens in which prior viral load testing was not done, both in the general analysis of all samples (aOR 0.64 95% CI: 0.13–0.56; p = 0.001) and even when DBS specimens were analysed alone (OR 0.68 95% CI: 0.6–0.77; p < 0.0001). It is possible that the specimens that were not subjected to pre-genotyping VL testing had VL < 5000 copies/mL, the range at which genotyping failure is more likely [22, 38]. Pre-genotyping viral load testing could be essential in qualifying specimens for HIVDR genotyping. Without pre-genotyping VL testing, time and resources may be wasted on genotyping non-eligible specimens resulting in low GSR and increasing the turn-around time. A study in Malawi reported that a delayed turnaround time in their laboratories was associated with lack of viral load testing and the use of DBS compared to plasma [53]. In our unpublished data, we noted that paediatric plasma and DBS samples with substantial genotyping failure turned out to be virally suppressed when we did viral load testing.

Despite the challenges of using plasma specimens in our setting, they still yield better genotyping success rates than DBS. The use of DBS specimens is a plausible option to plasma and whole blood due to its advantages which include the ease of collection, transportation, and storage. The WHO recommends the use of DBS specimens for viral load monitoring in resource-constrained settings where infrastructural and logistic deficiencies hinder the use of plasma specimens. Interestingly, a viral load threshold of ≥ 1000 copies/mL used to define virological failure with plasma specimens is also applicable to DBS specimens [7, 16]. Studies collaborate this with findings of high sensitivity and specificity of DBS specimens at a VL ≥ 1000 copies/mL [32, 54]. Any form of specimen that is used in HIVDR genotyping requires maximum effort towards ensuring that specimen collection, processing, transportation, storage, and testing is done appropriately so that accurate and reliable results are obtained.

This cross-sectional study is limited by several factors: The specimens analysed came from multiple sources, some of which were routine clinical diagnosis laboratories, which are not research-focused, thus, strict adherence to SOPs for proper specimen collection and handling cannot be guaranteed. Therefore, the widespread genotyping failure in most of the specimens from routine clinical diagnosis could have led to over-estimation of genotyping failure rates. Also, our analyses did not involve paired plasma and DBS specimens and as such our comparisons could have been influenced by other demographic and clinical variations in individuals from whom the specimens were collected. Despite these shortcomings, the findings herein remain applicable to other laboratories in our setting and this study will set precedence for further research into strategies of improving genotyping rates. It is also worth noting that our study strength was the use of a large sample size of specimens analysed in our laboratory and the inclusion of both samples from research studies and non-research focused sources. To the best of our knowledge, this is the first study in our setting to determine the laboratory genotyping success rates and examine the correlates of genotyping failure of unpaired DBS and plasma specimens from multiple studies/sources.

## Conclusions

We report decreasing overall HIVDR genotyping success rates with increased numbers of DBS specimens analysed between 2016 and 2019, for specimens collected in a resource-limited setting. While plasma specimens generally had higher GSR > 80% over this period, the GSR of DBS specimens decreased to as low as about 50%, compromising the genotyping performance of our laboratory. Genotyping failure correlated with the use of DBS specimens, low viral loads, lack of pre-genotyping viral load testing and the source of specimens being routine clinical diagnosis. The use of DBS specimens, in this resource-constrained setting, offers a viable alternative to plasma specimens for viral load testing and HIVDR monitoring. DBS is critical to the realisation of the ambitious UNAIDS 95-95-95 targets. We therefore recommend improvement in DBS handling, routine viral load testing prior to genotyping to screen samples with suppressed viral loads that are likely to fail genotyping tests. Highly sensitive assays should be developed to genotype specimens with low and undetectable viral load, especially in this era where VL suppression rates are rising following increased roll-out of ART.

## Data Availability

The datasets generated and analysed during the current study are not publicly available due to restriction policies and data protection policies of the MRC/UVRI & LSHTM but can be made available by the corresponding authors on reasonable request and on approval of the UVRI research ethics committee.
